# When sounds control sight: Associative learning modifies perceptual transitions in binocular rivalry

**DOI:** 10.1167/jov.26.3.2

**Published:** 2026-03-10

**Authors:** Stefano Ioannucci, Gabriel Leipner, Petra Vetter

**Affiliations:** 1 Visual and Cognitive Neuroscience Lab, Department of Psychology, University of Fribourg, Fribourg, Switzerland

**Keywords:** binocular rivalry, auditory conditioning, conscious perception, cross-modal interaction, associative learning, perceptual switches

## Abstract

Binocular rivalry occurs when incompatible images presented to each eye lead to alternations between two competing percepts. While several visual and multisensory factors can affect binocular rivalry dynamics, whether perceptual transitions themselves can be subject to cross-modal influences remains unknown. We developed a conditioning paradigm to test whether neutral auditory stimuli, when paired with visual probe–induced perceptual switches, could subsequently influence binocular rivalry dynamics. Participants viewed rivaling orthogonally oriented gratings of different colors. During conditioning, auditory stimuli were systematically paired with visual probes that triggered perceptual switches. Following conditioning, the presentation of conditioned sounds alone produced two effects: shorter dominance durations and, critically, faster perceptual switches. Control conditions confirmed that this conditioning effect could not be attributed to auditory stimulation itself, time on task, or report biases. Our findings provide evidence that binocular rivalry dynamics can be shaped by cross-modal associative learning processes, whereby conditioned sounds serve as predictive cues for perceptual transitions, effectively lowering the threshold for switches between competing stimuli. These results offer new insights into how auditory signals might be incorporated into predictive models that influence visual perception during the resolution of visual ambiguities.

## Introduction

In everyday vision, each of our eyes sees a slightly different version of the world, and both are seamlessly combined by the brain into a single, coherent, three-dimensional percept. This process, known as binocular fusion, allows us to perceive depth and navigate our environment with precision (Nelson, 1975). However, when the visual system is presented with substantially different images to each eye, binocular rivalry emerges. At any given moment during binocular rivalry, one image dominates conscious perception while the other is suppressed, followed by a “perceptual switch” where the previously suppressed image gains dominance. Occasionally, periods of mixed perception occur where elements of both images are simultaneously visible, referred to as “piecemeal” perception (Blake & O'Shea, 2009). Since 1838, when its main properties were scientifically described (Wheatstone, 1838), binocular rivalry has proven a valuable tool in perception research, due to its unique capacity to dissociate physical stimulation from conscious perception (Blake & Logothetis, 2002).

Regarding the neural mechanisms of binocular rivalry, several theoretical frameworks have been formulated. One proposes that rivalry primarily reflects competition between monocular neurons in early visual cortex. According to this eye-based account, inhibitory interactions between monocular channels lead to the suppression of one eye's input while the other eye's input dominates. Evidence for this view comes from studies showing that V1 activity is reduced during perceptual suppression (Lee & Blake, 2002) and that V1 activity in the monocular blind-spot representation positively correlates with perceptual dominance and suppression during rivalry (Tong & Engel, 2001). Other accounts propose that rivalry involves competition between higher-level stimulus representations rather than monocular channels, based on evidence that perceptual switches could occur independently of eye-of-origin information (Logothetis, Leopold, & Sheinberg, 1996; Leopold & Logothetis, 1999). More integrated perspectives suggest that rivalry involves multiple levels of visual processing, with competing interactions occurring at various stages of the visual hierarchy and beyond (Wilson, 2003; Freeman, 2005; Sterzer, Kleinschmidt, & Rees, 2009). From a computational perspective, predictive coding has been applied to explain binocular rivalry (Hohwy, Roepstorff, & Friston, 2008), proposing that the visual system might oscillate between competing signals while attempting to minimize prediction errors when faced with irreconcilable visual input.

These theoretical models are informed by the extensive work that has probed the susceptibility of rivalry dynamics to several mediators. For instance, early work studied how contrast affects perceptual dominance: Increasing the contrast of one stimulus primarily decreases the dominance duration of the other stimulus, while increasing the contrast of both stimuli increases the overall perceptual switch rate (Levelt, 1965). Similar effects have been observed for properties like luminance (Kakizaki, 1960) and spatial frequency (Fahle, 1982). Apart from these low-level visual stimulus properties, perceptual switches can be directly triggered through external interventions. Valle-Inclán, Hackley, de Labra, and Alvarez (1999) demonstrated that briefly presenting a probe stimulus (a small checkerboard pattern) to the suppressed eye could reliably induce a perceptual switch, making the suppressed image dominant and therefore reducing overall dominance durations. This probe-induced perceptual switching represents perhaps the most direct external manipulation of rivalry dynamics, providing a method to control the timing of perceptual transitions through a purely stimulus-based intervention. Further studies have characterized the spatial and temporal parameters that influence probe effectiveness (Mitchell, Stoner, & Reynolds, 2004; Metzger & Beck, 2020), showing that visual probes induce switches through a combination of enhancing the suppressed stimulus and suppressing the dominant one.

Beyond purely visual factors, cross-modal interactions, particularly between audition and vision, have been shown to influence binocular rivalry. Conrad, Bartels, Kleiner, and Noppeney (2010) found that directional motion sounds can enhance the dominance duration of congruently moving random-dot kinematograms during rivalry. Similarly, Chen, Yeh, and Spence (2011) demonstrated that natural sounds like bird calls or car engine noises can prolong the perceptual dominance of semantically matching images of birds and cars. Also, learned associations, rather than inherent ones, can alter rivalry dynamics. Wilbertz, van Slooten, and Sterzer (2014) demonstrated that reinforcement learning principles could be applied to binocular rivalry, showing that rewarded (or positively conditioned) gratings gained dominance, while punished (or negatively conditioned) gratings were suppressed. Einhäuser, Methfessel, and Bendixen (2017) examined how newly acquired audiovisual associations bias perception during rivalry by pairing rivaling gratings with distinct auditory stimuli (a pure 1000-Hz tone or pink noise). During a training phase, participants repeatedly experienced specific pairings of these stimuli, which subsequently increased dominance durations of their associated visual grating during rivalry. However, the associations created in these studies related to the stimuli, rather than the perceptual transitions themselves. The potential for specifically conditioning perceptual transitions to sounds, and thereby assessing their susceptibility to associative learning, has not been tested so far. Therefore, we hypothesized that pairing uninformative auditory cues repeatedly with visual probe-induced perceptual switches could subsequently influence rivalry dynamics when these cues were presented alone. Specifically, we predicted that after a conditioning phase, the presentation of a conditioned sound would lead to shorter visual dominance durations compared to preconditioning, demonstrating a learned audiovisual association with perceptual transitions.

## Methods

### Participants

A total of 88 (75 females, *M*_age_ = 22.26 ± 4.52) participants were recruited across two experiments and were compensated with psychology course credits or payment. All participants had normal or corrected-to-normal vision and were naive to the purpose of the study. Participants provided written informed consent prior to participation, and all procedures were approved by the Psychology Ethics Committee of the University of Fribourg.

### Apparatus

Visual stimuli were presented on an LCD monitor (ASUSTeK Computer, Taipei, Taiwan; resolution 1,024 × 768, refresh rate 60 Hz) viewed through a mirror stereoscope at a viewing distance of approximately 60 cm. A mirror stereoscope (ScreenScope; ASC Scientific, Narragansett, RI USA) was adjusted for each participant to ensure proper fusion of the two images to each eye. Head position was stabilized using a chin rest. Responses were collected via a standard computer keyboard. Sounds were presented via over-ear headphones (Sennheiser Electronic GmbH & Co, Wedemark-Wennebostel, Germany).

### Stimuli

**Figure 1.**
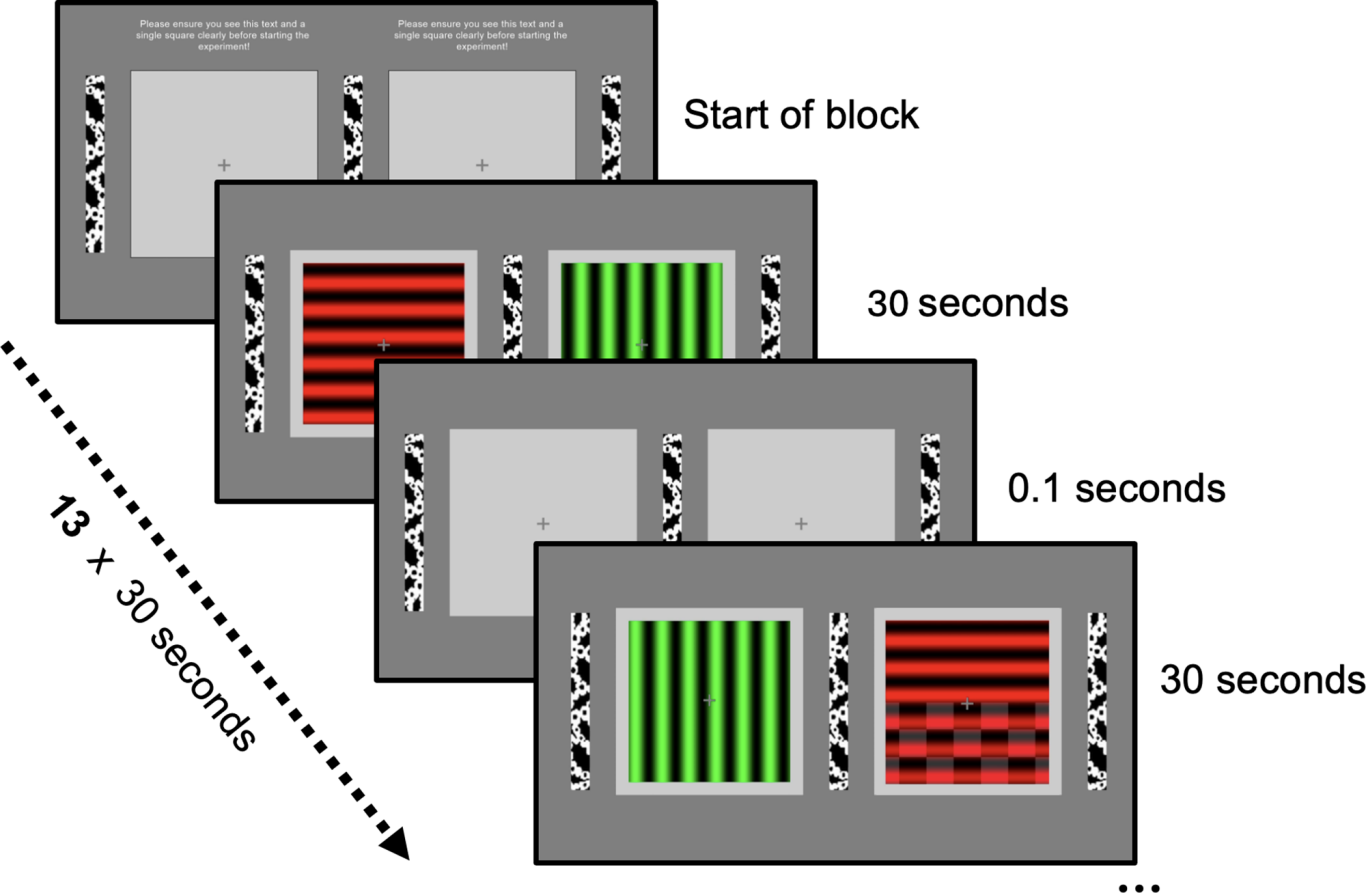
Experimental design and visual stimulus presentation. Each block began with a fusion check screen (top) displaying identical frames to both eyes, ensuring proper stereoscopic alignment. During experimental trials, participants viewed orthogonal gratings (horizontal/vertical) with different colors (red/green) presented to each eye in 30-second intervals. Between trials, a brief (500–700 ms) intertrial interval occurred with only fixation crosses visible. In subsequent trials, the orientation–color combinations were swapped between eyes. Throughout each trial, participants reported their dominant percept by pressing corresponding keys. In blocks that displayed a probe (Figure 2), a checkerboard pattern was flashed in the lower half of the suppressed stimulus (bottom right).

The main visual stimuli consisted of four different gratings (Figure 1), either horizontal or vertical black stripes on a red or a green background (2 × 2 design: horizontal/vertical orientation × red/green background). The gratings were presented in two squares of 14.5° × 12.1° on a gray background, one for each eye, fused by both eyes to a single square in the middle of the screen when viewed through a stereoscope. Visual probes consisted of black and white checkerboard patterns superimposed on the lower half of the suppressed grating according to the current perceptual dominance state. Visual probes were presented for 50 ms to the suppressed eye, with a 100-ms delay following each participant’s keypress, indicating a dominance report. The mean luminance, contrast, and saturation were constant across all stimuli. Depending on the experimental version, the auditory stimulus consisted either of a double sine wave tone composed of a pure 500 Hz tone (Pápai & Soto-Faraco, 2017) and a 1008 Hz tone (Einhäuser et al., 2017) of equivalent perceived loudness, as determined by the Fletcher–Munson curves (Experiment Version 1), or a brown noise sound (Experiment Version 2). Regardless of stimulus type, the sound was presented at approximately 65 dB sound pressure level (SPL) through closed-ear headphones in mono to avoid spatial sound cues. Auditory stimuli were presented concurrently with the visual probe onset and lasted for the 50-ms probe duration. In audio-only conditions, the same 50-ms auditory stimulus was triggered after a 100-ms delay following each keypress, indicating perceptual dominance. The tones were designed as simple sine waves without formants or spectral noise to avoid implicit associations with color (Anikin & Johansson, 2019).

### Procedure

**Figure 2.**
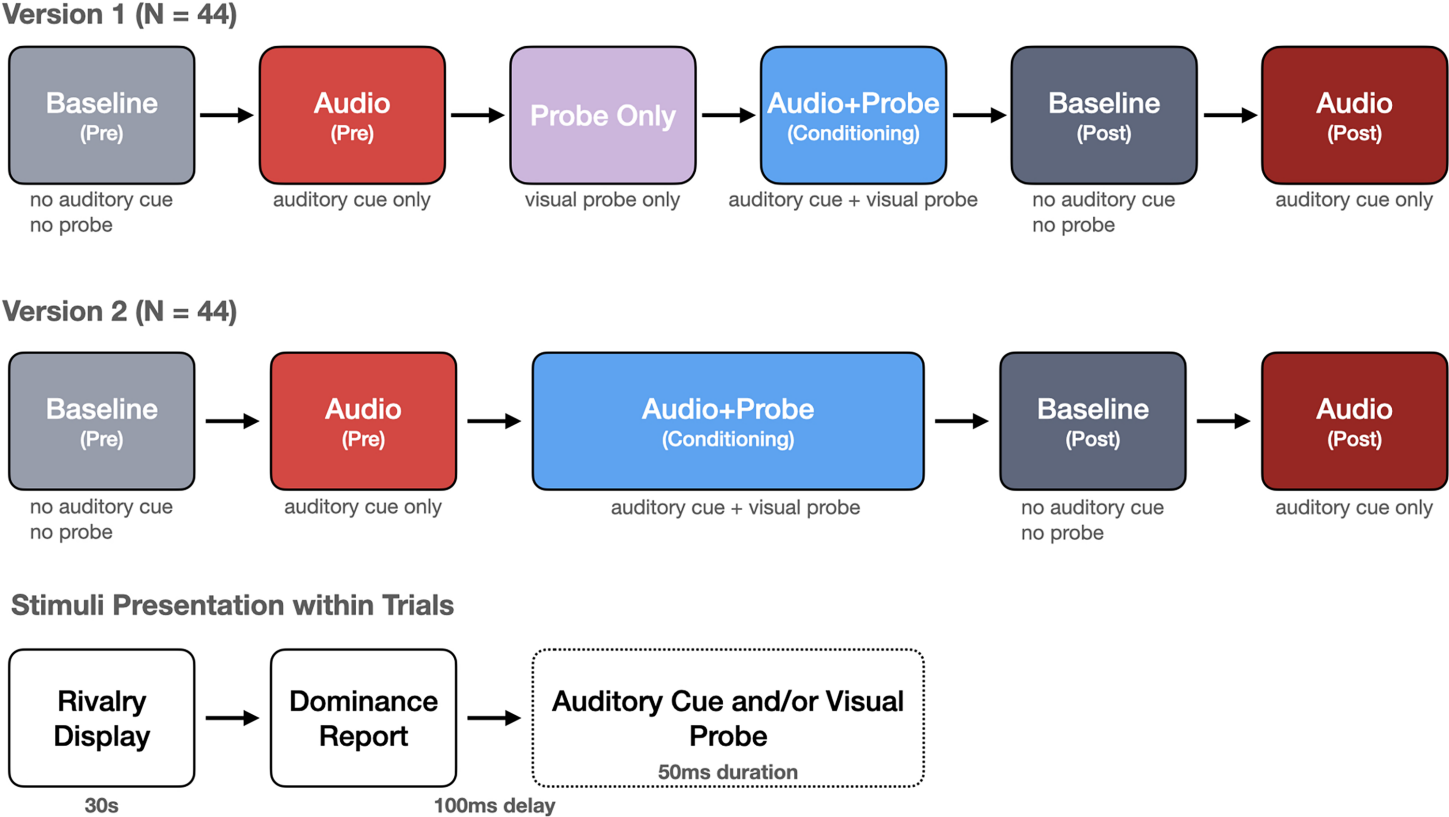
Experimental design and block structure. Top: Version 1 included six sequential blocks with a dedicated Probe Only condition to assess visual probe effectiveness independently. Middle: Version 2 omitted the Probe Only block and doubled the Audio + Probe conditioning duration, with partial (80%) auditory cue conditioning. Bottom: Trial structure within the blocks with additional stimulus presentation (nonbaseline). Following each keypress indicating perceptual dominance, a 100-ms delay preceded the simultaneous presentation of the auditory cue and/or the visual probe (checkerboard pattern) for 50 ms. The visual probe was presented to the currently suppressed eye to induce a perceptual switch. Each block comprised 12 trials of 30 seconds each. Block order for Baseline and Audio conditions was counterbalanced in both pre- and postconditioning phases in both experimental versions. Version 2 also included catch trials (one per block) in which identical stimuli were presented to both eyes.

The experiment was divided into several blocks, each corresponding to a specific experimental condition (Figure 2). In each block, participants viewed a random combination of two of the four main gratings (one in each eye), with the constraint that the gratings always differed in both color and orientation (one red and one green; one with vertical stripes and one with horizontal stripes). This combination changed pseudorandomly for each 30-second trial, with a brief intertrial interval randomly varying between 500 and 700 ms between trials. The four gratings appeared with equal frequency throughout the experiment.

The experiment began with a short training phase to familiarize participants with the task. Data from this phase were not included in the analyses. Throughout all blocks, participants were instructed to report “whether the main percept was green or red” by pressing and holding one of two keys on a keyboard with their right hand, one key when they perceived the red grating and another when they perceived the green grating. When their perception was mixed (piecemeal), participants were instructed not to press any key. Dominance phases were defined as the time intervals between keypress and key release.

The experiment consisted of six sequential blocks. The “Baseline Pre” block featured binocular rivalry without visual probes or auditory stimuli to measure baseline dominance durations. In the “Audio Pre” block, auditory tones were presented coinciding with participants’ key presses to determine whether the mere addition of an auditory stimulus would affect perceptual reports. For Version 1 only (see below for experimental variations; Figure 2), a “Probe Only” block was included where visual probes (checkerboard patterns) were presented superimposed on the lower half of the suppressed grating, according to the current dominance report. The conditioning block “Audio + Probe” featured both the auditory tone and visual probe presented simultaneously, with the probe shown only on top of the suppressed grating. This block served to associate the tone with probe-enforced perceptual switches. Following conditioning, a “Baseline Post” block presented binocular rivalry without probes or auditory stimuli (Similar to the Baseline Pre block), and the final “Audio Post” block featured auditory tones co-occurring with key presses (similar to the Audio Pre block). This block was the key experimental condition to investigate whether the auditory stimulus alone could alter rivalry dynamics after conditioning.

Each block consisted of 12 thirty-second trials for a total of 6 minutes per block. The entire experiment, including training, lasted approximately 60 minutes, with voluntary short breaks provided between blocks to mitigate fatigue and eye strain.

### Experimental variations

To thoroughly investigate our hypotheses and ensure robust findings, we conducted two variants of the basic paradigm:

**Version 1** (*N* = 44) implemented the full six-block design described above. This version included a dedicated Probe Only block to assess the effectiveness of visual probes alone in triggering perceptual switches. The order of the Baseline and Audio blocks was counterbalanced between participants in both the preconditioning and postconditioning phases. In this version, the auditory stimulus consisted of a double sine wave tone (500 Hz and 1008 Hz) presented, coinciding with every dominance report during the Audio + Probe conditioning block.
**Version 2** (*N* = 44) was preregistered (https://osf.io/xu3qz) and designed to strengthen the conditioning effect by omitting the Probe Only block and doubling the duration of the Audio + Probe conditioning block. This version also included an additional catch trial in each block (six per participant). Catch trials were included to assess whether participants showed systematic response biases unrelated to perceptual dominance. On catch trials, identical stimuli (same color and orientation) were presented to both eyes, eliminating binocular rivalry. Under these conditions, participants should perceive a stable, unambiguous image and report minimal perceptual switching. Elevated switch rates on catch trials would indicate random or inattentive responding rather than genuine perceptual reports. As in Version 1, the order of the Baseline and Audio blocks was counterbalanced between participants in both preconditioning and postconditioning phases. In this experiment, brown noise bursts were employed as auditory stimuli, and a partial reinforcement schedule was implemented where the sound was presented coinciding with 80% of dominance reports during conditioning.

### Terminology

Dominance duration refers to the time interval between key press and key release for each perceptual state (i.e., how long a single percept was continuously reported).

Perceptual switch refers to a transition between mutually exclusive dominance reports (i.e., a change from reporting red to green or vice versa).

Cue-to-switch latency refers to the time elapsed between auditory cue onset and a subsequent perceptual switch, used to characterize the temporal dynamics of conditioning effects.

### Data preprocessing

The primary dependent variable was dominance duration, defined as the time interval between key press and key release for each perceptual state (reporting either the green or red percept). To ensure robust analysis, we identified and removed outlier dominance durations for each participant under each experimental condition, following the interquartile method (Leys, Ley, Klein, Bernard, & Licata, 2013), amounting to 4.4% of total reports (Supplementary Figure S1).

For each experimental trial, we calculated the average dominance duration by taking the mean of all valid (nonoutlier) dominance durations.

We also examined individual participant data for anomalous patterns across conditions. Based on visual inspection of the data, one participant (Version 2; not included in the sample description) was excluded because their dominance durations showed an extreme deviation from those of all other participants, with values more than three times the group average in the postconditioning blocks (Supplementary Figure S2).

To directly measure the temporal relationship between conditioned sounds and perceptual dynamics, we recorded the latency between sound presentation and subsequently reported perceptual switches. For each trial in the blocks with auditory stimuli (Audio Pre and Audio Post), we calculated the average time elapsed between stimulus onset and reported perceptual switches indicated by a change in button press. This provided a direct measure of how quickly after its onset the auditory stimulus could trigger a perceptual switch.

### Statistical analysis

All analyses were conducted using linear mixed-effects models implemented in R with the lme4 package. For the pooled analysis (*N* = 88), we modeled each trial's average dominance duration as a function of block type (fixed effect) with random intercepts and random slopes for block type by participant:

$$\begin{array}{ccc} & & avg\;dominance\;duration∼block\;type\\ & & \;+\left(1+block\;type|participant\right) \end{array}$$

For version-specific analyses, only random intercepts were used given the smaller sample size:

$$\begin{array}{c} \;avg\;dominance\;duration∼block\;type+\left(1|participant\right) \end{array}$$

Multiple comparisons were corrected using the Holm–Bonferroni method unless otherwise specified. Bayesian paired *t*-tests with default JZS priors (*r* = 0.707) were conducted to quantify evidence for null effects where relevant. To verify that methodological differences between experimental versions did not compromise pooling, we compared participant-level conditioning effects (Audio Post minus Audio Pre) between versions using an independent-samples *t*-test (*T*_(__86)_ = −0.89, *p* = 0.38, BF₁₀ = 0.31). A random-effects meta-analysis of the version-specific estimates further confirmed the robustness of the pooled effect (*p* = 0.002). We therefore present the pooled analysis in the main text; full model contrast coefficients are reported in Supplementary Tables S1 to S3.

Our primary analyses focused on testing whether auditory stimuli could be conditioned to influence binocular rivalry dynamics. Specifically, we examined whether after conditioning (a) dominance durations in the Audio Post condition would decrease compared to Audio Pre, indicating less stable rivalry dynamics elicited by the conditioned tones; (b) the Baseline condition would show no differences between pre- and postmeasurements; and (c) a decrease of dominance durations would emerge between Audio Post and Baseline Post conditions that was not present in the preconditioning phase (i.e., Audio Pre vs. Baseline Pre).

Additionally, we performed several exploratory and control analyses: To determine whether conditioning affected perceptual switches versus perceptual states, we quantified each participant's dominance bias toward either the green or the red stimulus across all experimental conditions. For each participant and condition, we calculated the total duration spent perceiving red versus green stimuli, defining bias as (red dominance – green dominance)/(red dominance + green dominance), with positive values indicating red preference and negative values indicating green preference. We then labeled each participant's percept as “preferred” (matching their overall bias) or “nonpreferred” (opposite to their bias). The conditioning effect (difference in average dominance duration between pre- and postconditioning) was calculated separately for preferred and nonpreferred percepts and compared using a paired-samples *t*-test.

To examine how conditioned auditory cues influenced the timing of perceptual switches, we analyzed switches occurring within 3 seconds of cue onset in the Audio Pre and Audio Post conditions. Perceptual switches were identified from continuous key press data as transitions between mutually exclusive dominance reports, with switch time defined as the moment of the second key press. We employed two complementary analyses to characterize temporal dynamics. First, to identify when conditioning effects occurred, we divided the 3-second postcue window into 30 consecutive 100-ms bins and calculated the switch probability for each bin. Statistical significance was assessed using within-subject permutation testing (1,000 iterations) where pre/post condition labels were randomly swapped within each participant, generating a null distribution that preserved the paired structure. The *p*-values were computed for each time bin and corrected for multiple comparisons using false discovery rate correction. Second, to quantify the magnitude of the conditioning effect, we calculated each participant's immediate switch rate, defined as the proportion of perceptual switches that followed an auditory cue within 500 ms. These rates were compared between Audio Pre and Audio Post conditions using a paired-samples *t*-test.

The effectiveness of the checkerboard probe in inducing perceptual switches was quantified by calculating, for each probe presentation, whether a perceptual switch (change in reported dominant percept) occurred within a 3-second window following probe onset. Probe effectiveness was defined as the proportion of probe presentations that resulted in a perceptual switch within this time window, calculated separately for Probe Only and Audio + Probe conditions.

To rule out the possibility that the reported main effect reflected temporal expectancy or the natural rise in switch probability over time (baseline hazard) rather than genuine conditioning effects, we conducted an event history analysis. We fit a Cox proportional hazards model (Cox, 1972) to individual dominance durations in the Audio blocks to account for baseline hazard. We additionally tested whether conditioning effects varied with position within trial (defined as the *N*th dominance report, from first to last) by including this as a covariate in a mixed-effects model restricted to the Audio Pre and Audio Post blocks with the following formula:

$$\begin{array}{ccc} & & \;dominance\;duration\;∼\;block\;type\;+\;position\;+\;block\;type\\ & & \;\;\times position+\left(1+block\;type|participant\right) \end{array}$$

Finally, we also binned dominance periods by terciles into early (<7th report), middle (<13th report), and late (<*N*th report) trial positions and tested the conditioning effect within each bin in separate linear mixed models.

For Version 1, we also explored differences in dominance times between the Probe Only and Audio + Probe conditions to test additive effects of sound on the consequences of visual probes. For Version 2, we analyzed responses to catch trials across block types to assess whether participants showed systematic response biases, unrelated to dominant percepts, in their reporting behavior that might influence the main results.

## Results

**Figure 3.**
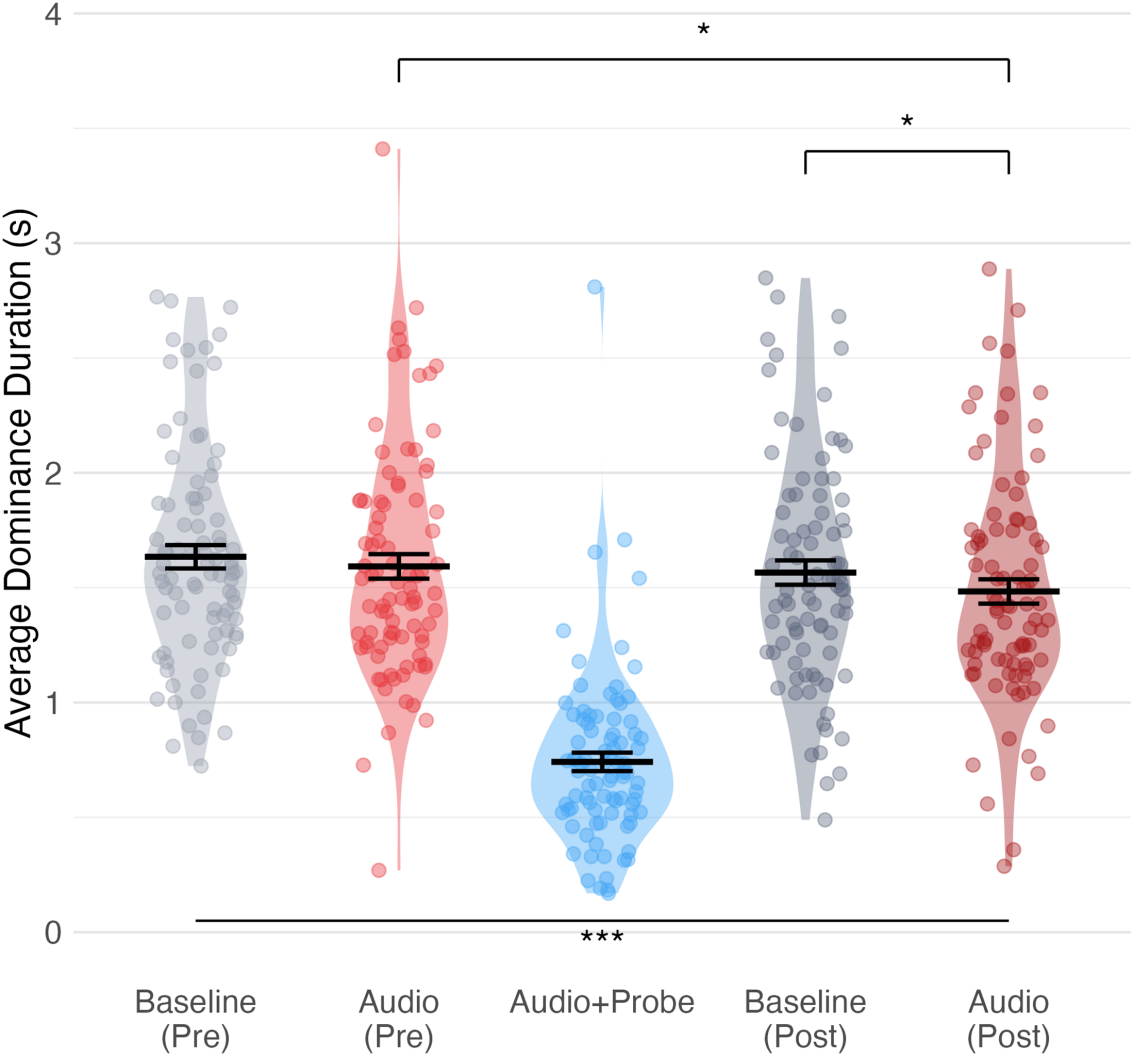
Average dominance duration per participant across experimental conditions. Light gray: Baseline preconditioning. Light red: Audio preconditioning. Blue: Audio + Probe (conditioning phase). Dark gray: Baseline postconditioning. Dark red: Audio postconditioning. Horizontal bars depict the group means, while error bars represent the standard error of the mean. *p_corr_ < 0.05, ***p_corr_ < 0.001. Statistical comparisons with Holm-corrected *p*-values are reported in Supplementary Table S3.

We first conducted an overall analysis of variance on the mixed-effects model to determine whether the experimental manipulations had an effect on dominance durations. The results revealed a significant main effect of block type (χ²_(4)_ = 386, *p* < 0.001), indicating that dominance durations varied across the different phases of the experiment (Figure 3).

Baseline rivalry dynamics were consistent with typical binocular rivalry (Blake, 2001). In the Baseline Pre condition, mean dominance duration was 1.6 ± 0.5 seconds, and participants reported an average of 16.3 perceptual transitions per 30-second trial. Analysis of keypress sequences confirmed that genuine perceptual switches (transitions between different percepts) comprised 95.2% of all transitions, with return transitions (same percept reported after brief interruption) representing only 4.8% of cases.

Having established an overall main effect of experimental condition, we proceeded with planned contrasts to test our specific hypotheses regarding the conditioning effects of auditory stimuli on binocular rivalry dynamics.

First, we verified that no difference was observed between Audio Pre and Baseline Pre conditions (β = 0.04, *SE* = 0.03, *Z* = 1.7, *p*_corr_ = 0.13, Cohen's *d* = 0.1), indicating that presentation of a sound per se was not sufficient to modulate dominance reports. To examine the effect of conditioning perceptual switches with tones, we compared pre- and postmeasurements in the audio condition. The contrast revealed a significant decrease in dominance durations from Audio Pre to Audio Post (β = −0.11, *SE* = 0.04, *Z* = −2.76, *p*_corr_ = 0.02, *d* = 0.21). This reduction supports our hypothesis that conditioning sounds with perceptual switches leads to shorter dominance durations when sounds are subsequently presented alone compared to when the sound is unrelated to perceptual switches.

To determine whether this effect was specific to the audio condition rather than a general effect of time or fatigue, we compared the pre- and postmeasurements in the baseline condition. The contrast showed a nonsignificant decrease between Baseline Pre and Baseline Post (β = −0.07, *SE* = 0.04, *Z* = −1.84, *p*_corr_ = 0.13, *d* = 0.12). Critically, we found a significant difference between Baseline Post and Audio Post conditions (β = −0.08, *SE* = 0.03, *Z* = −2.87, *p*_corr_ = 0.02, *d* = 0.17), with shorter dominance durations in the Audio Post condition. This difference confirms that the conditioned auditory stimulus decreased dominance durations more than any general time-related changes. The Audio + Probe conditioning phase itself showed the shortest dominance durations across all conditions (all *p*s < 0.001), which was expected given the known impact of the visual probe on perceptual dynamics (Valle-Inclan et al., 1999). The shorter dominance durations in this phase compared to all other conditions confirmed the effectiveness of the probe in triggering perceptual switches, which was essential for establishing the conditioning effect. To complement our analyses and address potential concerns about interpreting nonsignificant results, we conducted Bayesian paired *t*-tests with default JZS priors (*r* = 0.707) on participant-averaged dominance durations. This analysis provided negligible evidence for the null hypothesis (no difference) in the Baseline Pre and Baseline Post contrast (BF₁₀ = 0.57) and in the Audio Pre versus Baseline Pre contrast (BF₁₀ = 0.45), while highlighting meaningful changes in the Audio Pre versus Audio Post contrast (BF₁₀ = 4.1) and in the Baseline Post versus Audio Post contrast (BF₁₀ = 5.3).

To confirm that these effects were not attributable to temporal expectancy or rising baseline hazard, we conducted multiple control analyses (Supplementary Figure S6). The Cox proportional hazards model revealed that dominance periods in the Audio Post condition showed an elevated hazard of resulting in a switch compared to Audio Pre, even after accounting for baseline hazard (hazard ratio = 1.17, 95% CI [1.14, 1.19], *p* < 0.0001). Furthermore, the mixed model including dominance reports within trial position as a covariate maintained significance for the conditioning effect (β = −0.1, *SE* = 0.04, *Z* = −2.8, *p*_corr_ = 0.014), with the interaction not being significant (β = 0.001, *SE* = 0.001, *Z* = 0.64, *p*_corr_ = 0.52). Moreover, stratified analysis by position terciles revealed significant conditioning effects in early (β = −0.11, *SE* = 0.04, *Z* = −2.8, *p*_corr_ = 0.018) and middle (β = −0.1, *SE* = 0.04, *Z* = −2.4, *p*_corr_ = 0.034) trial positions, with a smaller, nonsignificant effect for late positions (β = −0.05, *SE* = 0.04, *Z* = −1.5, *p*_corr_ = 0.14). Collectively, these analyses demonstrate that the conditioning effect operates independently of temporal factors. It persists when controlling for baseline hazard, does not interact with the position within a trial, and is present from the earliest dominance reports within each trial rather than emerging as temporal expectations accumulate.

Concerning participants’ dominance bias for one versus the other grating, the average change in dominance duration between Audio Pre and Audio Post conditioning blocks was −0.11 ± 0.37 seconds for the preferred percept and −0.10 ± 0.37 seconds for the nonpreferred percept, with no significant difference across participants (*T*_(87)_ = −0.36, *p* = 0.73). This independence from perceptual states strongly supports the idea that the audio cues were conditioned to the switch process itself, rather than biasing perception toward a particular perceptual state.

**Figure 4.**
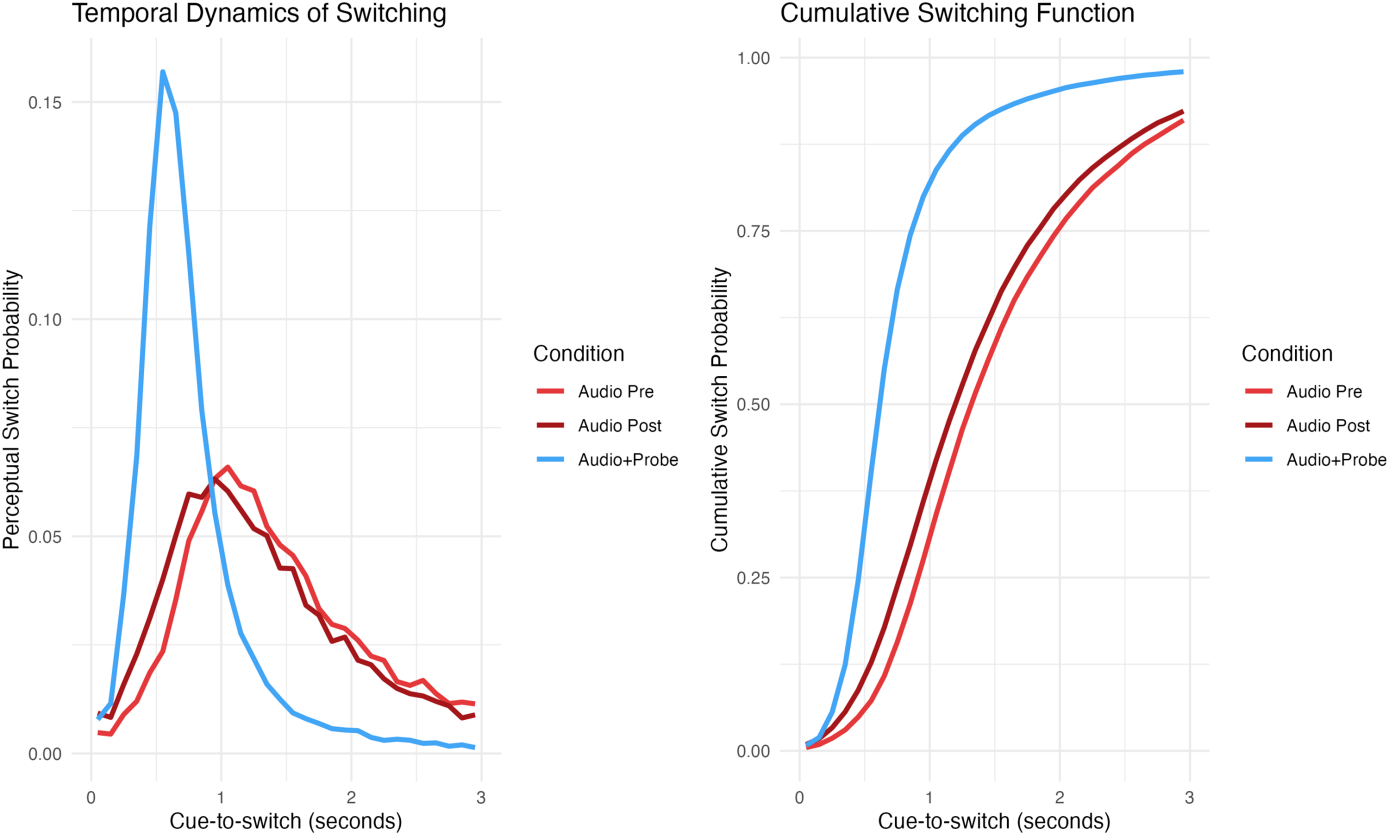
Temporal dynamics of perceptual switches following auditory cue presentation. Left panel: Distribution of perceptual switch probability as a function of cue-to-switch latencies, showing the proportion of switches occurring in each 100-ms time bin. Right panel: Cumulative switch probability as a function of cue-to-switch latencies, showing the proportion of total switches that had occurred by each time point; steeper slopes indicate faster switching. Light red: Audio Pre (before conditioning). Dark red: Audio Post (after conditioning). Blue: Audio + Probe (conditioning phase).

Analysis of switch timing revealed that conditioning produced a temporally specific enhancement of perceptual transitions (Figure 4). Within-subject permutation testing identified a significant increase in switch probability confined to the time window of 300 to 600 ms postcue (*p*_corr_ < 0.05), with the effect peaking at 500 to 600 ms. To quantify this effect at the participant level, we examined immediate switch rates (<600 ms postcue). These increased significantly from 5.5% ± 4.2% in Audio Pre to 9% ± 8.3% in Audio Post conditions (*T*_(__87)_ = 4.87, *p* < 0.0001, *d* = 0.52), representing a 60% relative increase. The convergence of both analyses demonstrates that conditioned cues transiently accelerated imminent perceptual switches rather than artificially disrupting rivalry dynamics or increasing overall switch frequency.

Concerning the effectiveness of the visual probe in inducing perceptual switches, we confirmed that it showed high effectiveness, inducing switches in 88% of presentations in the Probe Only condition and 91% in the Audio + Probe condition. Both conditions showed peak switch probability between 500 and 700 ms postprobe (Probe Only: 625 ms; Audio + Probe: 525 ms), with no significant differences between them (paired *t*-test; *T*_(__43)_ = −1.1, *p* = 0.28).

To assess if the auditory stimulus itself contributed to the effectiveness of the visual probe during the conditioning phase, we compared dominance durations between the Probe Only and Audio + Probe conditions (Supplementary Figure S3). This analysis included only the subset of participants from Version 1 (*N* = 44). A linear mixed-effects model with random slopes for block type revealed no significant difference in dominance durations between the Probe Only and Audio + Probe condition (β = −0.03, *SE* = 0.02, *Z* = −1.1, *p* = 0.28, *d* = 0.1). A Bayesian paired *t*-test confirmed these null findings (BF₁₀ = 0.29). The uninformative sound, therefore, had no particular impact on the magnitude of the effect of the visual probe, serving as a temporal marker that became associated with perceptual switches rather than directly influencing dominance durations.

To verify that our main findings reflected genuine perceptual effects rather than response biases, we analyzed performance on catch trials in which identical stimuli were presented to both eyes. This analysis included only the subset of participants from Version 2. Data screening identified one participant whose average dominance duration in the Audio + Probe condition (1.39 seconds) was more than 3 standard deviations below the group mean (*M* = 4.58 seconds, *SD* = 0.7 seconds; Supplementary Figure S4). This extreme value likely resulted from a misunderstanding of instructions, with the participant potentially releasing response keys when seeing the probe despite no change in dominant percept. Therefore, this participant's data for the Audio + Probe block were removed from this specific analysis.

Catch trial mean dominance duration was 4.7 ± 0.6 seconds with 1.6 ± 0.3 switches on average. Therefore, participants clearly distinguished rivalry from nonrivalry conditions, with substantially longer dominance durations and fewer reported switches on catch trials.

Concerning the mixed model, the overall analysis of variance revealed a significant effect of block type (*F*_(4, 211)_ = 2.8, *p* = 0.025). However, the planned contrasts examining conditioning effects showed no significant differences between pre- and postconditioning phases after correction for multiple comparisons (Audio Pre vs. Baseline Pre: β = 0.06, *SE* = 0.11, *T*_(__214)_ = 0.59, *p*_corr_ = 1, *d* = 0.17; Audio Pre vs. Post: β = −0.22, *SE* = 0.11, *T*_(__214)_ = −2.04, *p*_corr_ = 0.17, *d* = 0.33; Baseline Pre vs. Post: β = −0.02, *SE* = 0.11, *T*_(__214)_ = 0.2, *p*_corr_ = 1, *d* = 0.05; Audio Post vs. Baseline Post: β = −0.14, *SE* = 0.11, *T*_(__214)_ = 1.26, *p*_corr_ = 0.62, *d* = 0.2). Bayesian paired *t*-tests further confirmed support for the null hypothesis in the Baseline Pre versus Baseline Post contrast (BF₁₀ = 0.17), while providing marginal evidence for a difference in the Audio Pre versus Audio Post contrast (BF₁₀ = 1.4). These results indicate that there was a temporary reduction in dominance durations, particularly in the Audio + Probe conditioning phase itself, highlighting a tendency for diverging effects in the control and conditioned blocks that did not result in prominent and lasting changes (Supplementary Figure S5, Supplementary Table S4)—in any case, negligible compared to the robust conditioning effect observed in rivalry trials, thereby supporting our interpretation that the conditioning effect reflects a genuine perceptual effect rather than solely response biases or task demand characteristics. To further assess whether generic response tendencies contributed to the conditioning effect, we correlated catch trial performance with conditioning effect magnitude. Neither catch trial dominance duration (*r* = 0.22, *p* = 0.16), catch trial switch rate (*r* = 0.003, *p* = 0.98), nor baseline rivalry dominance (*r* = −0.08, *p* = 0.6) correlated significantly with the magnitude of the conditioning effect. This dissociation indicates that individual differences in response tendencies do not account for the observed conditioning.

## Discussion

The present study investigated whether auditory stimuli could be conditioned to influence perceptual transitions in binocular rivalry. Our results provide evidence that multisensory associative learning can affect the dynamics of conscious perception during binocular rivalry. After pairing neutral sounds with visual probe–induced perceptual switches during a conditioning phase, those same sounds subsequently produced shorter dominance durations and accelerated subsequent perceptual switches, compared to preconditioning. Moreover, the difference between audio and baseline conditions emerged only after conditioning, confirming that the sounds acquired their influence through associative learning rather than through the mere addition of auditory cues or the consequences of time on task. Importantly, these effects could not be attributed to temporal confounds. Event-history analyses confirmed that the conditioning effect persisted after controlling for baseline switching hazard and did not interact with position within trial. If temporal expectancy had driven our results, effects should have strengthened across successive dominance periods within a trial as participants accumulated exposure to the sound-switch contingency. Instead, conditioning effects were significant for early and middle positions but attenuated for late positions, suggesting that the effect reflects genuine associative learning established during conditioning rather than within-trial expectancy building up from repeated cue presentations.

Our findings extend previous research on cross-modal influences and associative learning in binocular rivalry, highlighting how cross-modal associative processes can influence what has often been characterized as a purely visual, low-level competitive process. While earlier studies demonstrated that congruent sounds can enhance the perceptual dominance of matching visual stimuli (Conrad et al., 2010; Chen et al., 2011) and that sounds or rewards could be associated with specific stimuli to bias perception toward them (Einhauser et al. 2017; Wilbertz et al. 2014), our study focused specifically on conditioning sounds to the perceptual transitions themselves rather than to static percepts. Here, we found that meaningless sounds can affect perceptual dynamics without being associated with any single competing visual stimulus, suggesting that the mechanisms underlying cross-modal influences on rivalry are more flexible and adaptive than previously recognized. This supports hierarchical neural models that incorporate both bottom-up and top-down influences, in which competition in binocular rivalry occurs at multiple processing levels simultaneously, with feedback connections allowing higher-level or cross-modal associations to influence early visual processing. Recent neurophysiological evidence from animals further strengthens this hierarchical interpretation. Garner and Keller (2021) established that learned audiovisual associations can reshape cortical circuits in mouse primary sensory cortex, with conditioned auditory cues producing systematic suppression of visual responses in layer 2/3 V1 neurons. This suppression effect is particularly relevant for binocular rivalry, as its role was recently highlighted in macaque V1 as a potential trigger for perceptual switches (Carlson et al., 2023).

Our results suggest a possible mechanism: During conditioning, the simultaneous presentation of sounds and probe-induced perceptual switches may create a statistical learning opportunity whereby the sound becomes associated with perceptual transitions. The auditory cue might then function as a learned predictor that increases readiness for perceptual transitions, potentially lowering the threshold for switch initiation. While speculative in the absence of direct neuroimaging data, one candidate pathway could involve feedback projections from multisensory integration areas (Macaluso & Driver, 2005) to early visual cortex. However, we emphasize that this neural account remains hypothetical and would require convergent evidence from neuroimaging or neurophysiological studies to substantiate.

From a predictive coding perspective, binocular rivalry reflects competing error-minimization processes. When the prediction error associated with the suppressed stimulus accumulates beyond a threshold, a perceptual switch occurs to resolve the discrepancy between perceived and suppressed stimuli (Hohwy et al., 2008). Our findings are consistent with the possibility that through conditioning, the auditory cue may become a learned predictor of imminent perceptual transitions, effectively lowering the threshold required for a switch to occur. This interpretation is supported by the temporal dynamics of the observed effect, where switches that would have occurred later are “pulled forward” in time by the cue, explaining why we observed reduced dominance durations without a dramatic sustained peak in switch probability.

While we demonstrate that conditioned sounds can influence rivalry dynamics, some caveats must be addressed. First, we cannot definitively distinguish between direct conditioning of sounds to perceptual switches and indirect conditioning to the expectation of the visual probe itself. That is, the conditioned sound may have become associated with the checkerboard probe rather than the perceptual switch per se, subsequently triggering expectations that facilitate switches through top-down mechanisms. However, several aspects of our data suggest the conditioning operated at a genuine perceptual level rather than pure probe expectancy. For instance, the temporal dynamics show the conditioned effect confined to 300 to 600 ms postcue, matching the timing of actual probe-induced switches in our data (median ∼500 ms). Additionally, the effect persisted across hundreds of Audio Post trials despite no accompanying probe. If participants simply anticipated a probe that never came, we would expect rapid extinction of the conditioned effect that we did not observe. Lastly, our catch trial analysis revealed minimal conditioning effects when identical stimuli were presented to both eyes, despite the same temporal pairing of sounds and probes. This indicates that the conditioning requires actual binocular rivalry and perceptual competition, rather than simply reflecting learned stimulus–response associations or probe-related expectations. Future studies could more definitively test this distinction by varying probe properties between conditioning and test phases or by employing noncontingent sound–probe pairing.

Second, the learned effect was, somewhat predictably, smaller than that of direct visual probes. Thus, internally conditioned auditory stimuli may have more limited access to visual perceptual switching mechanisms than the high-contrast visual probe that is capable of enforcing perceptual switches. Further studies may elucidate whether more extensive conditioning protocols might strengthen this effect, employing objective measures beyond self-report to confirm the perceptual nature of the conditioning effect we observed.

Third, it may be argued that the relatively large stimulus size employed here could have potentially increased mixed percept duration or led to problematic “return” transitions where participants report the same dominant percept after brief interruptions. However, as reported, baseline rivalry dynamics were typical (Blake, 2001), and return transitions were rare.

Taken together, our results show that perceptual switches during binocular rivalry can be influenced by conditioned auditory stimuli, providing evidence for the flexible nature of conscious perception through associative learning mechanisms. These findings demonstrate that cross-modal associative processes can influence basic perceptual dynamics, expanding our understanding of how the brain resolves visual ambiguity and selects information for conscious awareness. Notably, the conditioning paradigm developed in this study offers a novel method for manipulating perceptual states during bistable perception without direct visual stimulation, which may be valuable for further investigations. Moreover, if perceptual switches can indeed be conditioned, this could provide a new noninvasive therapeutic approach in applied vision science based on the principles of associative learning.

## Supplementary Material

Supplement 1

Supplement 2

Supplement 3

Supplement 4

Supplement 5

Supplement 6

Supplement 7

Supplement 8

Supplement 9

Supplement 10
