# Supplementary figures and images for: When sounds control sight: Associative learning modifies perceptual transitions in binocular rivalry
Source: J Vis. 2026 Mar 10;26(3):2. doi: 10.1167/jov.26.3.2 (PMC13001832; doi:10.1167/jov.26.3.2)

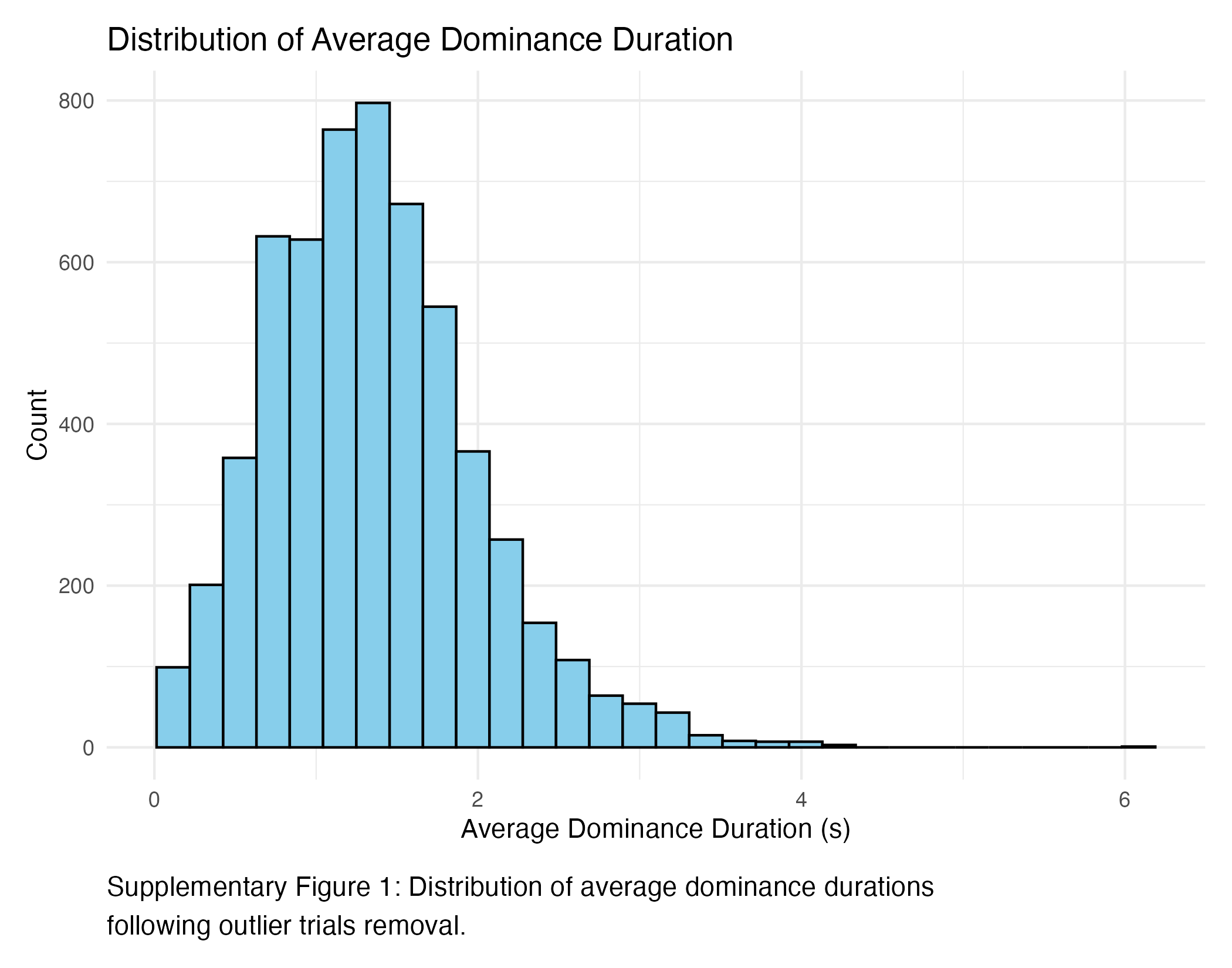

Supplement: Supplement 1 [file jovi-26-3-2_s001.tiff]

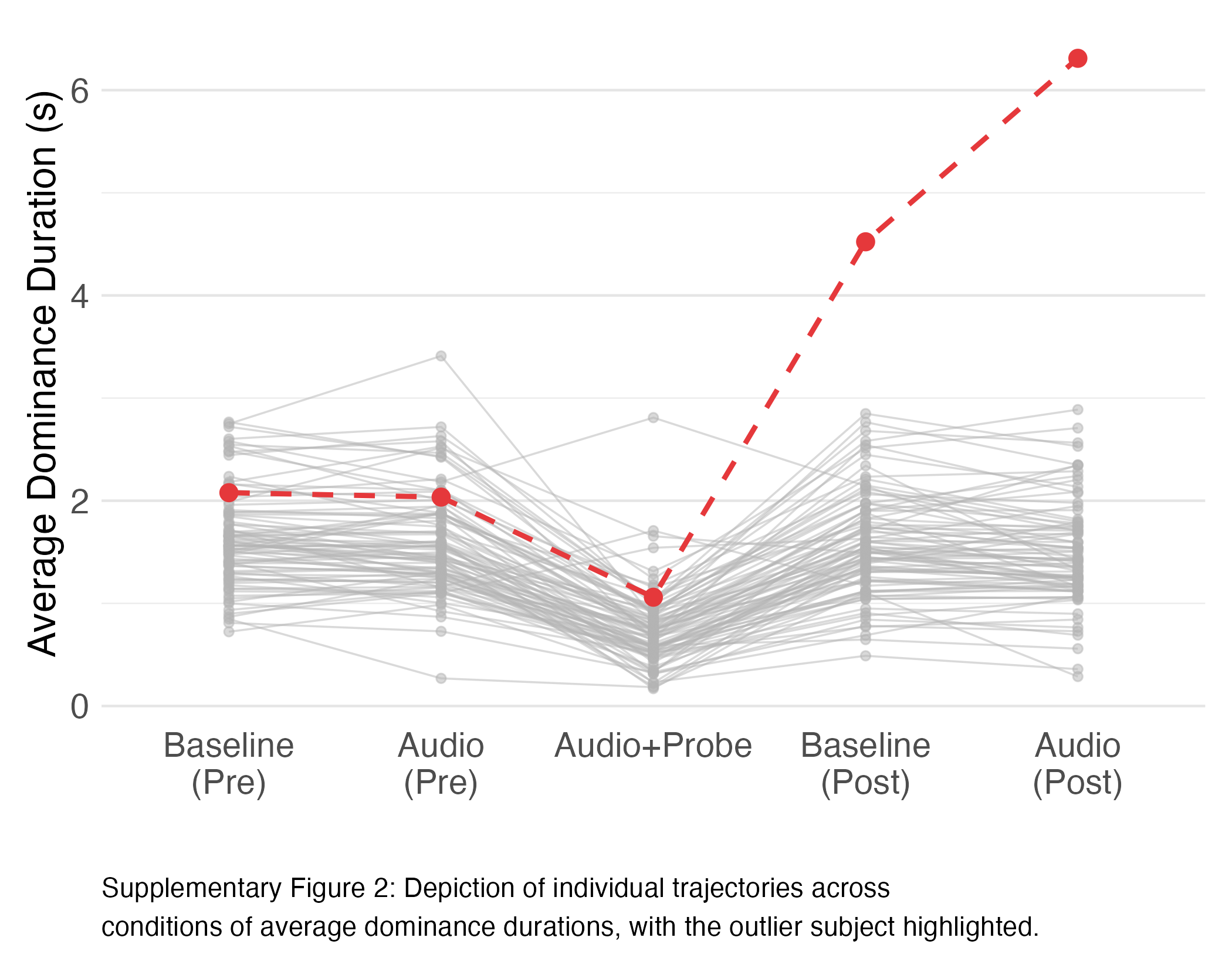

Supplement: Supplement 2 [file jovi-26-3-2_s002.tiff]

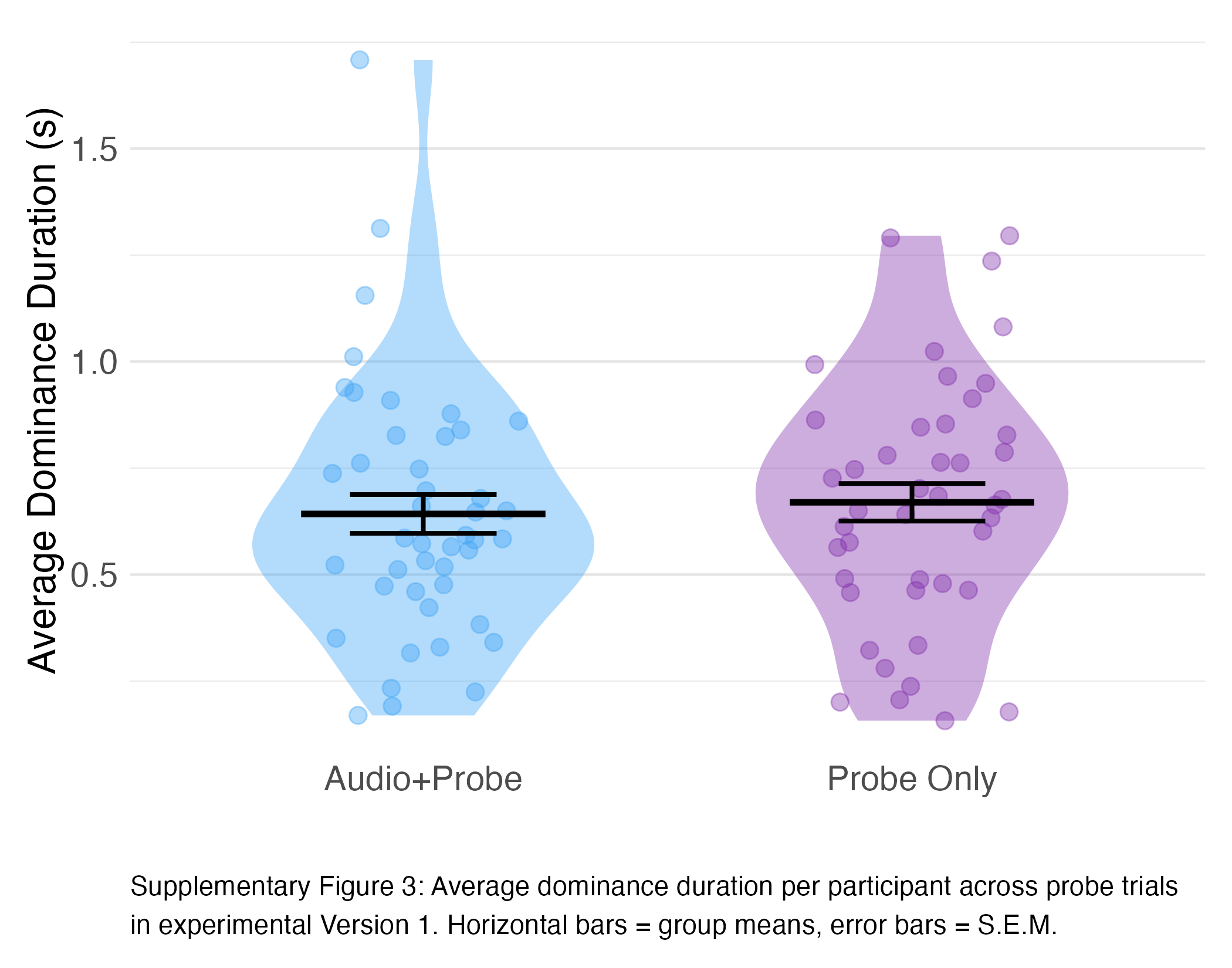

Supplement: Supplement 3 [file jovi-26-3-2_s003.tiff]

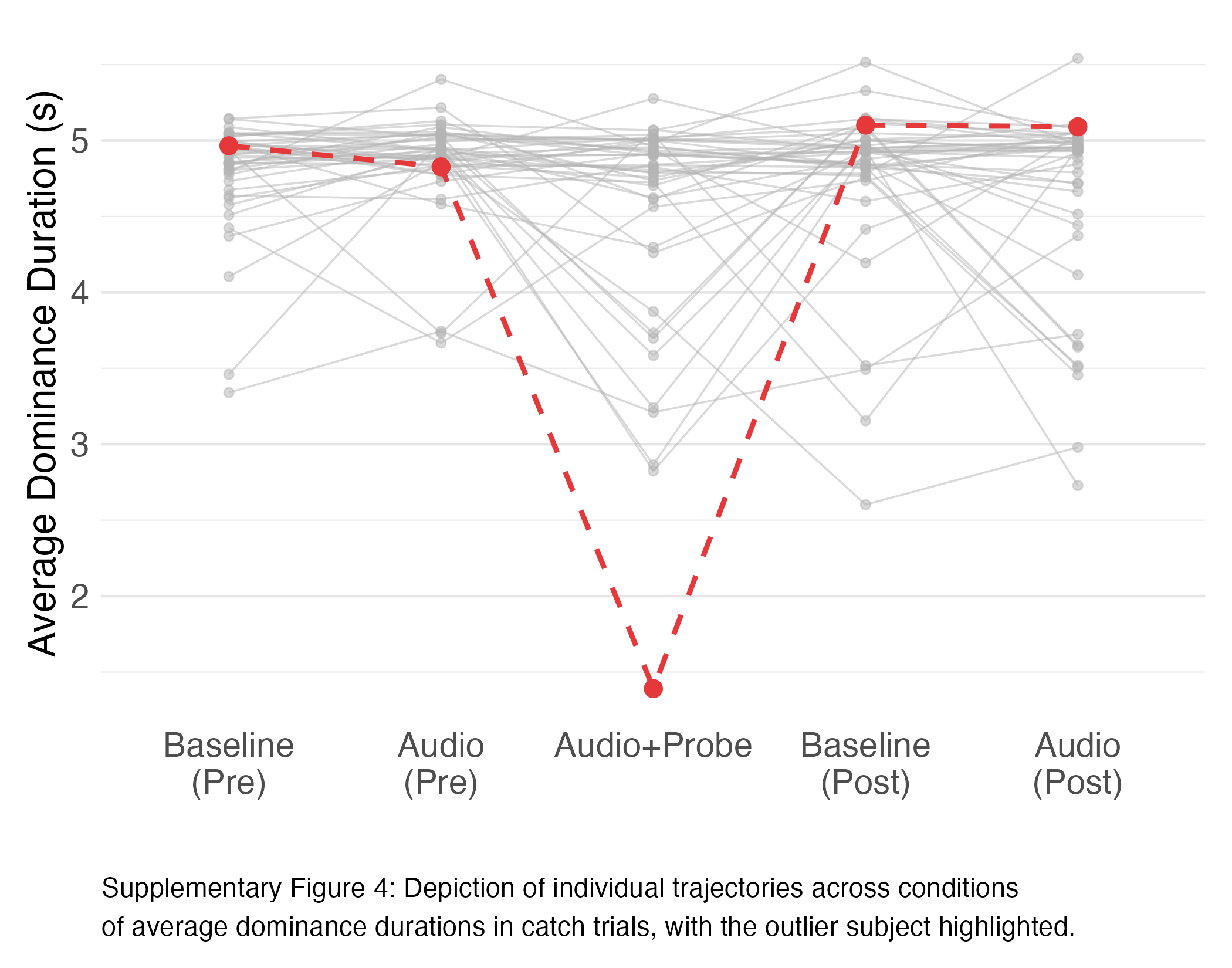

Supplement: Supplement 4 [file jovi-26-3-2_s004.tiff]

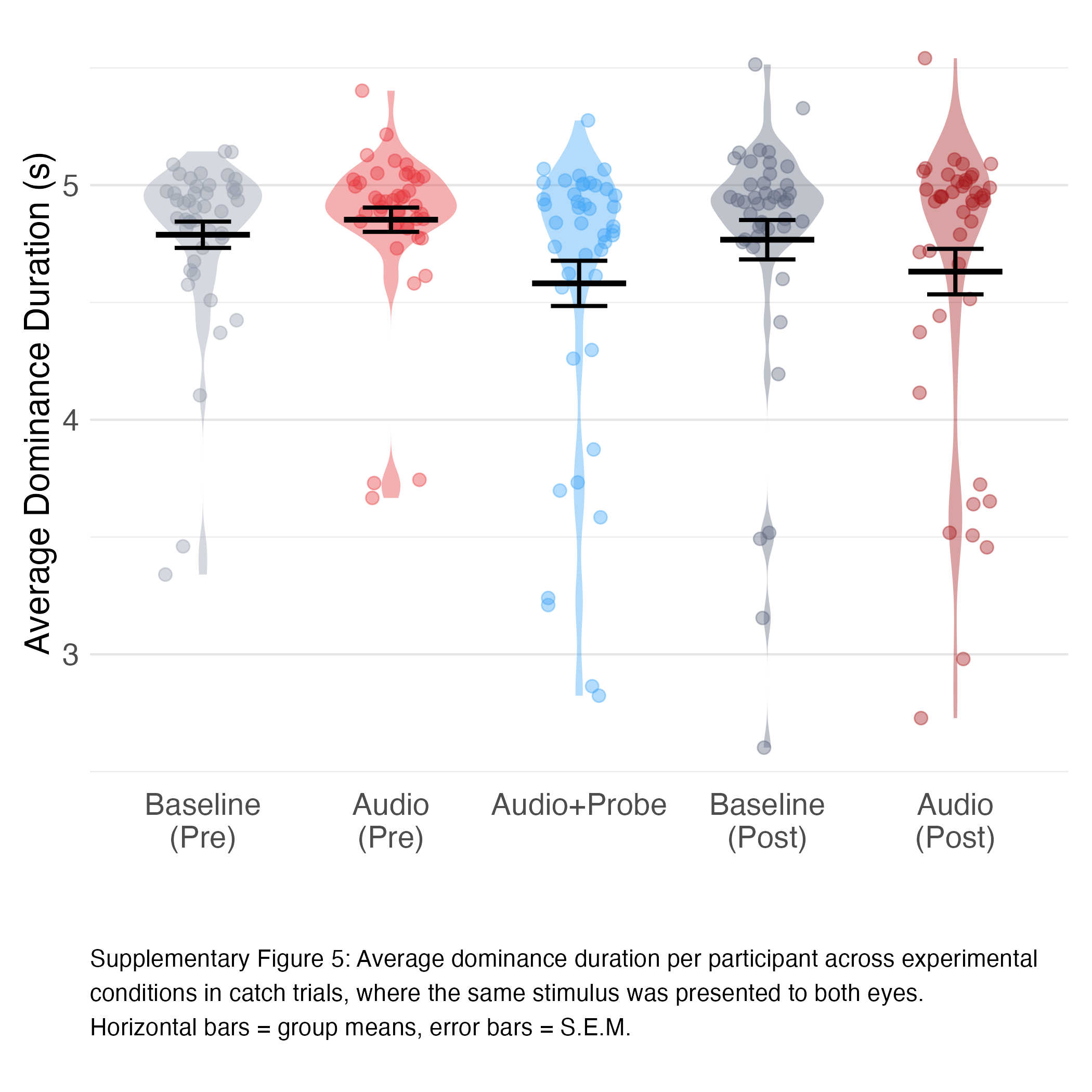

Supplement: Supplement 5 [file jovi-26-3-2_s005.tiff]

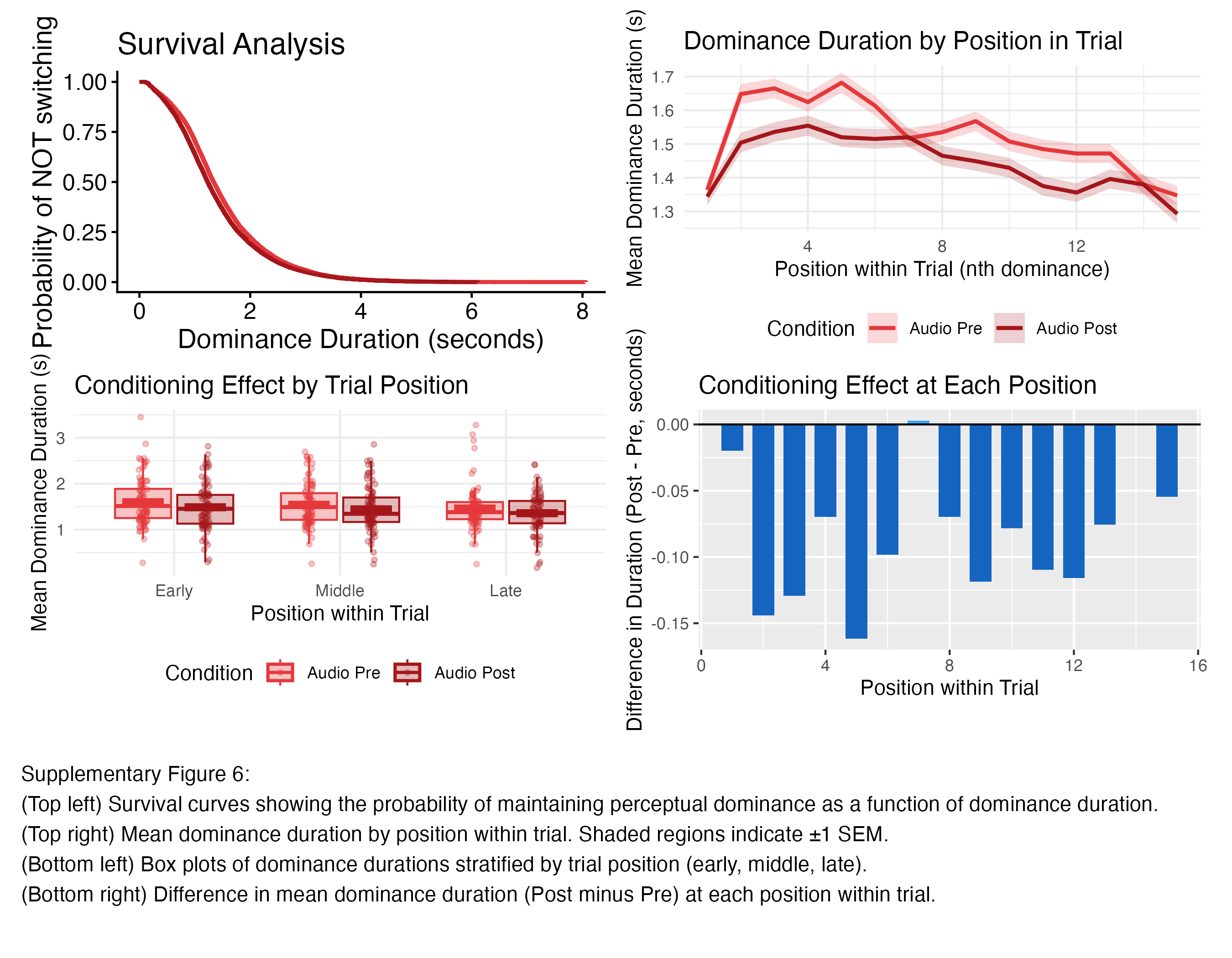

Supplement: Supplement 6 [file jovi-26-3-2_s006.tiff]
