# Supplementary material for: When sounds control sight: Associative learning modifies perceptual transitions in binocular rivalry
Source: J Vis. 2026 Mar 10;26(3):2. doi: 10.1167/jov.26.3.2 (PMC13001832; doi:10.1167/jov.26.3.2)
Supplement: Supplement 7 [file jovi-26-3-2_s007.pdf]

Supplementary Table 1 - Version 1 Model

model: average dominance ~ block type + (1 | subject)

Fixed Effects (reference: Baseline Pre)

Parameter	Estimate
Intercept	1.615
Audio Pre	–0.048
Audio+Probe	–0.975
Baseline Post	–0.12
Audio Post	–0.192

Random Effects

Component	Std. Dev.
Intercept (Subject)	0.387
Residual	0.373

Contrasts

Contrast	Estimate	SE	95% CI	p (Holm)
Audio Post – Audio Pre	–0.145	0.023	[–0.19, –0.1]	<0.0001
Baseline Post – Baseline Pre	–0.12	0.023	[–0.166, –0.075]	<0.0001
Audio Pre – Baseline Pre	–0.048	0.023	[–0.093, –0.003]	0.039
Audio Post – Baseline Post	–0.072	0.023	[–0.117, –0.027]	0.004
