# Supplementary material for: When sounds control sight: Associative learning modifies perceptual transitions in binocular rivalry
Source: J Vis. 2026 Mar 10;26(3):2. doi: 10.1167/jov.26.3.2 (PMC13001832; doi:10.1167/jov.26.3.2)
Supplement: Supplement 8 [file jovi-26-3-2_s008.pdf]

Supplementary Table 2 - Version 2 Model

model: average dominance ~ block type + (1 | subject)

Fixed Effects (reference: Baseline Pre)

Parameter	Estimate
Intercept	1.658
Audio Pre	–0.04
Audio+Probe	–0.817
Baseline Post	–0.018
Audio Post	–0.114

Random Effects

Component	Std. Dev.
Intercept (Subject)	0.39
Residual	0.317

Contrasts

Contrast	Estimate	SE	95% CI	p (Holm)
Audio Post – Audio Pre	–0.073	0.022	[–0.117, –0.029]	0.003
Baseline Post – Baseline Pre	–0.019	0.022	[–0.063, 0.025]	0.404
Audio Pre – Baseline Pre	–0.04	0.022	[–0.084, 0.004]	0.141
Audio Post – Baseline Post	–0.095	0.022	[–0.139, –0.051]	0.0001
