# Supplementary material for: When sounds control sight: Associative learning modifies perceptual transitions in binocular rivalry
Source: J Vis. 2026 Mar 10;26(3):2. doi: 10.1167/jov.26.3.2 (PMC13001832; doi:10.1167/jov.26.3.2)
Supplement: Supplement 9 [file jovi-26-3-2_s009.pdf]

Supplementary Table 3 - Pooled (Version 1 + Version 2) Model

model: average dominance ~ block type + (1 + block type | subject)

Fixed Effects (reference: Baseline Pre)

Parameter	Estimate
Intercept	1.635
Audio Pre	–0.043
Audio+Probe	–0.893
Baseline Post	–0.069
Audio Post	–0.152

Random Effects

Component	Std. Dev.
Intercept (Subject)	0.469
Audio Pre	0.206
Audio+Probe	0.454
Baseline Post	0.336
Audio Post	0.364
Residual	0.275

Contrasts

Contrast	Estimate (s)	SE	95% CI	p (Holm)
Audio Post – Audio Pre	–0.109	0.04	[–0.188, –0.031]	0.02
Baseline Post – Baseline Pre	–0.069	0.038	[–0.144, 0.005]	0.14
Audio Pre – Baseline Pre	–0.043	0.025	[–0.092, 0.007]	0.14
Audio Post – Baseline Post	–0.082	0.029	[–0.139, –0.025]	0.02
