# Supplementary material for: When sounds control sight: Associative learning modifies perceptual transitions in binocular rivalry
Source: J Vis. 2026 Mar 10;26(3):2. doi: 10.1167/jov.26.3.2 (PMC13001832; doi:10.1167/jov.26.3.2)
Supplement: Supplement 10 [file jovi-26-3-2_s010.pdf]

Supplementary Table 4 - Catch Trial Model (Version 2)

model: average dominance ~ block type + (1 | subject)

Fixed Effects (reference: Baseline Pre)

Parameter	Estimate
Intercept	4.789
Audio Pre	0.064
Audio+Probe	–0.204
Baseline Post	–0.021
Audio Post	–0.157

Random Effects

Component	Std. Dev.
Intercept (Subject)	0.261
Residual	0.507

Contrasts

Contrast	Estimate (s)	SE	95% CI	p (Holm)
Audio Pre – Audio Post	–0.221	0.11	[–0.45, –0.014]	0.17
Baseline Pre – Baseline Post	0.021	0.11	[–0.242, 0.192]	1
Audio Pre – Baseline Pre	0.064	0.11	[–0.148, 0.286]	1
Audio Post – Baseline Post	–0.136	0.11	[–0.356, 0.078]	0.63
